# Old adults preserve motor flexibility during rapid reaching

**DOI:** 10.1007/s00421-017-3584-2

**Published:** 2017-03-14

**Authors:** Christian Greve, Tibor Hortobágyi, Raoul M. Bongers

**Affiliations:** 1 Center for Human Movement Science, University of Groningen, University Medical Center Groningen, Hanzeplein 1, HPC CB41, Postbus 30.001, 9700 RB Groningen, The Netherlands; 2 Department of Rehabilitation Medicine, Center for Rehabilitation, University Medical Center Groningen, University of Groningen, Groningen, The Netherlands

**Keywords:** Motor control, Ageing, Motor flexibility, Uncontrolled manifold, Reaching, Task demand

## Abstract

**Purpose:**

Our ability to flexibly coordinate the available degrees of freedom allows us to perform activities of daily living under various task constraints. Healthy old adults exhibit subclinical peripheral and central nervous system dysfunctions, possibly compromising the flexibility in inter-joint coordination during voluntary movements and the ability to adapt to varying task constraints.

**Method:**

We examined how healthy old (75.4 ± 5.2 years, *n* = 14) compared with young adults (24.3 ± 2 years, *n* = 15) make use of the available motor flexibility to adapt to physical and dexterity constraints during a rapid goal-directed reaching task. We manipulated physical and dexterity demands by changing, respectively, external resistance and target size. Motor flexibility was quantified by an uncontrolled manifold (UCM) analysis.

**Results:**

We found that healthy young and old adults employ similar motor flexibility as quantified by the ratio between goal equivalent and non-goal equivalent variability (*V*_Ratio_) and were similarly able to adapt to increases in physical and dexterity demands during goal-directed rapid reaching (*V*_Ratio_: *p* = .092; young: 0.548 ± 0.113; old: 0.264 ± 0.117). Age affected end-effector kinematics. Motor flexibility and end-effector kinematics did not correlate.

**Conclusion:**

The data challenge the prevailing view that old age affects movement capabilities in general and provide specific evidence that healthy old adults preserve motor flexibility during a reaching task. Future studies applying UCM analysis should examine if experimental set-ups limit movement exploration, leaving possible age differences undetected.

## Introduction

Environmental challenges and task constraints require the neuromuscular system to adapt when performing goal-directed actions in daily life. Flexibility is a prominent feature of motor adaptability. Flexibility is the capacity of the neuromuscular system to make fine adjustments in the coordination of the available degrees of freedom (e.g., joint angles) while ensuring successful completion of the intended motor act (Domkin et al. 2005; Latash et al. 2007; Scholz and Schöner 1999). However, even healthy old adults exhibit subclinical peripheral and central nervous system dysfunctions such as increased agonist–antagonist muscle co-activation, deterioration in the size and number of muscle fibers, and impaired intracortical inhibition, possibly affecting motor flexibly (Bassey et al. 1992; Beijersbergen et al. 2013; Faulkner et al. 2007; Ge et al. 2002; Goble et al. 2009; Hortobágyi and Devita 2006; Pantoni 2002; Papegaaij et al. 2014; Peinemann et al. 2001; Romanovsky et al. 2015; Schulz et al. 2014; Thompson 2009).

Predictably, old compared with young adults execute activities of daily living (ADLs) more slowly, unsteadily, and inaccurately (Bock 2005; Bock and Girgenrath 2006; Buch et al. 2003; Heuer and Hegele 2008; McNay and Willingham 1998; Seidler 2006). However, there is conflicting evidence as to how and if at all old age affects motor flexibility during reaching, as motor flexibility during reaching was less (Dutta et al. 2013; Verrel et al. 2012), similar (Xu et al. 2013), or even greater (Krüger et al. 2013) in old compared with young adults. Moreover, old compared to young adults employ less motor flexibility during multi-finger force coordination (Kapur et al. 2010; Olafsdottir et al. 2007; Shinohara et al. 2004) and standing balance tasks (Hsu et al. 2013, 2014), but similar motor flexibility during the initiation of a step over an object (Wang et al. 2015), walking (Krishnan et al. 2013) and hand rotation tasks (Skm et al. 2012) and larger motor flexibility in sit-to-stand tasks (Greve et al. 2013).

We address two factors as potential sources of the inconsistencies in age differences in motor flexibility during goal-directed reaching movements. First, previous studies reported similar total movement time (Verrel et al. 2012; Xu et al. 2013) and peak velocity (Xu et al. 2013) during reaching in old and young participants. These similarities between age groups contrast with the age-related decrease in reaching performance and might represent behavior at the boundary of the natural motor repertoire possibly biasing motor flexibility in favor of old adults (Sleimen-Malkoun et al. 2013; Van Halewyck et al. 2015). Second, previous studies did not manipulate task constraints during reaching even though reaching is done under a variety of conditions in daily life. Thus, Aim 1 was to determine the effects of age on the use of the available motor flexibility while performing goal-directed reaching under physical and dexterity constraints. We hypothesized that healthy old compared with young adults use less motor flexibility, select fewer of the available joint configurations, and reduce performance stability with increasing demands. Considering the inconclusive effects of age on motor flexibility, our alternative hypothesis is that motor flexibility is retained in old age despite reductions in movement velocity. To further understand how healthy ageing affects reaching behavior, Aim 2 examined the association between end-effector kinematics (i.e., reaching speed and accuracy on target) and motor flexibility in each age group.

## Methods

### Participants

Healthy young (4 males and 11 females, 24.3 ± 2 years) and old (4 males and 10 females, 75.4 ± 5.2 years) adults participated in the study. Participants were free of neurological or musculoskeletal disorders in the neck, shoulder, arm or hand and had normal or corrected to normal vision.

### Experimental design

Participants performed a goal-directed upper extremity reaching task as fast and accurately as possible under four experimental conditions: low dexterity and low physical demand, low dexterity and high physical demand, high dexterity and low physical demand, and high dexterity and high physical demand. In a control experiment, healthy young adults performed the same reaching task but at a comfortable instead of maximal speed. We manipulated physical demand by increasing the resistance to the reaching movement expressed as 0% (low; 0.2 kg) and 13% (high) of the averaged maximum voluntary contraction (MVC) values. We selected the 13% load based on pilot data (*n* = 4) and kinematic data from a previous study (Greve et al. 2015). We manipulated dexterity demand by decreasing the diameter of the target while the distance between start position and target location was kept constant [low: 1.56 cm (= index of difficulty (ID): 4) and high: 0.39 cm (ID: 6)] (Fitts 1954). Before the start of each reaching condition participants performed three familiarization trials using the new weight or dexterity condition. After familiarization, participants performed 25 reaching trials for each condition. The order of reaching conditions for each block of 25 trials was randomized between participants. In total each participant performed 4 conditions of each 25 reaching trials, resulting in 100 reaching movements.

### Experimental set-up

**Fig. 1:**
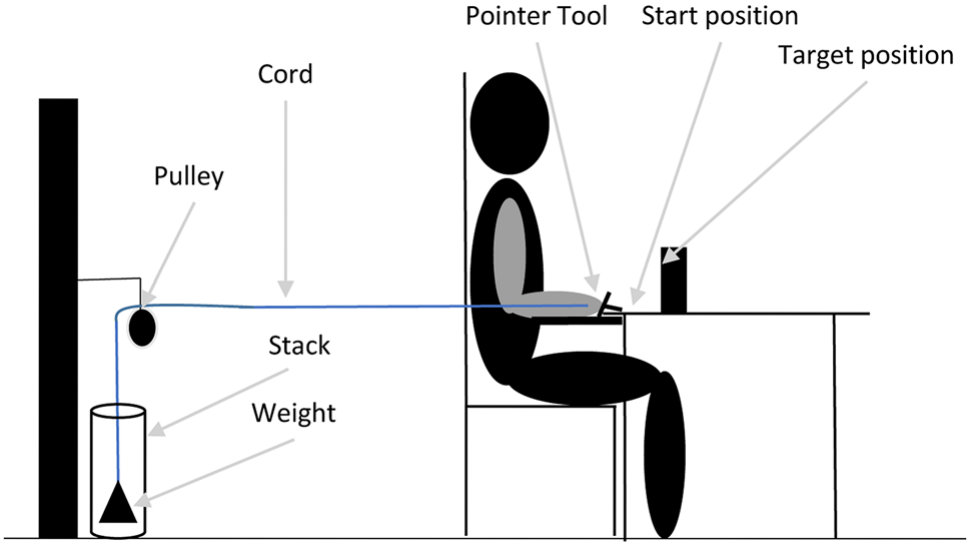
Experimental set-up. Participants sat in an adjustable chair in front of a table so that the olecranon process with the elbow flexed at 90° was at tabletop height. The start posture was approximately 20° shoulder abduction, 90° elbow flexion and 90° pronation. To have a consistent start position, participants placed their right olecranon on an elbow support and the pointer tip in a pre-defined start position. The elbow support was positioned at the right side of the participants’ body at the same height with the table. The start position of the pointer tip was marked on the table with a dot of the size of the diameter of the pointer tip. During the start position the back of the pointer tool was placed against a wooden bar to release the load (Greve et al. 2015)

Figure 1 shows a schematic overview of the experimental set-up described in detail previously (Greve et al. 2015). Participants reached with a small handheld tool in a forward direction toward a target. A cord connected the handheld tool and a pulley that could hold small weights. During the reaching movement the cord was situated between the participant’s arm and trunk without interfering with the reaching movement. An Optotrak motion capture system consisting of two units recorded at 100 Hz the position of six triangular rigid body markers each equipped with an LED in each of the three corners that were used to collect the data. The rigid bodies were attached on the sternum (legs of the triangle were 6 cm), and on the right side of the body on the acromion (legs of the triangle were 3 cm), on the lateral aspect of the upper arm just proximal to the insertion of the deltoid muscle (legs of the triangle were 6 cm), the lateral aspect of the lower arm just proximal to the ulnar and styloid process (legs of the triangle were 6 cm), the dorsal surface of the hand (legs of the triangle were 3 cm) and at the pointer tool (legs of the triangle were 3 cm) (van Andel et al. 2008). A crossover harness minimized trunk movement during reaching. Before each reaching trial, the experimenter checked and corrected, if needed, the start position of the participants. The distance between start position of the pointer tip and the target location was 25 cm (18.8 cm in depth and 16.6 cm vertical distance from table top).

### Experimental procedures

First, we measured body mass, height, and handedness (Oldfield 1971). To define the weight for the 13% physical demand condition and to quantify whether participants fatigued during the experiment, before and after the experiment participants performed three trials of 4-s-long MVCs against a load cell of a hand-held dynamometer (ErgoFet, Hoggan Health Industries, West Jordan, USA). The MVC was executed in the start position of the reaching movement (Fig. 1). The average of the three trials was used in the analysis. Participants rested for 30–60 s between consecutive MVCs. In the 0% weight condition a weight of 0.2 kg was attached to the cord, which was just enough to keep the cord taut.

In the main experiment, the young and old adults reached toward the target as fast as possible in response to an auditory cue. After each reaching movement, participants remained in contact with the target until a second auditory cue and then moved the arm back to the start position. The experimenter emphasized that the reaching task was not a reaction time task, but that after initiation of the reaching movement the participants had to move as fast as possible. At any time during the experiment participants were allowed to pause, rest, and drink water. There was one minute of rest between conditions. The entire experiment lasted 45–60 min, including putting on the markers and calibrating the system.

### Joint angle computation

Joint angles were computed based on standards established by the International Society of Biomechanics (Wu et al. 2005). Based on bony landmarks and the displacements of the markers on the rigid bodies, local coordinate systems were computed. Global and local orientations of segment coordinate systems were calculated based on the combination of the local coordinate systems (Wu et al. 2005). During the calibration procedure, 17 bony landmarks were digitized with a standard pointer device (van Andel et al. 2008). Based on the joint position data, we computed the following joint rotations: shoulder plane elevation, shoulder elevation, shoulder inward–outward rotation, elbow flexion–extension, forearm pronation–supination, wrist flexion–extension and wrist abduction–adduction.

### UCM analysis

To establish age differences in motor flexibility, we subjected the joint position data to UCM analysis. The UCM method decomposes trial-to-trial variability in the effector system into variability stabilizing task important variables (e.g., end-effector position during reaching), coined goal equivalent variability (GEV), and variability causing a deviation of the end-effector from its desired position [non-goal equivalent variability (NGEV)] (Latash et al. 2007; Tseng et al. 2003). GEV underlies co-variation between joints of the moving limb and reflects variability in joint configuration patterns with which the end-effector position is not affected. Our UCM analysis was performed at the end-point of the reaching movement for the UCM components GEV, NGEV and the ratio between GEV/NGEV (*V*_Ratio_), with the latter indicating the strength of the stabilizing effect of motor flexibility on the end-effector position. We hypothesized that GEV would be lower and NGEV similar in old as compared to young adults. UCM analysis was based on the covariance matrix C and performed as described in detail previously (Latash et al. 2007; Yen and Chang 2010; Verrel 2011). The joint angular data of the shoulder, elbow and wrist were used as elemental variables resulting in a 7-DOF system. The pointer tip position (2 DOF) was selected as the performance variable. The Jacobian (J), necessary to relate changes in elemental variables to changes in the performance variable, was computed based on a 3D forward kinematics model relating joint configurations to pointer tip position (Domkin et al. 2005; van der Steen and Bongers 2011). The accuracy of the forward kinematics model was tested previously (Greve et al. 2015).

### Individual joint variability and multi-joint covariation

The UCM analysis performed as described above does not differentiate between UCM effects due to individual joint variability or multi-joint covariation. To establish whether UCM effects originated from multi-joint covariation and were not confounded by individual joint variability, a permutation analysis was performed. We performed permutation analysis based on the covariance matrix C (Verrel 2011; Yen and Chang 2010). The covariance matrix of the permuted data set (C_Perm_) was computed by setting the off-diagonal terms to zero. GEV_Perm_ and NGEV_Perm_ were computed as the original UCM components but then with the covariance matrix C_Perm_. As a measure for the amount of UCM effects originating from individual joint variability we computed the ratio between GEV_Perm_ and NGEV_Perm_ (*V*_RatioPerm_). Larger amounts of *V*_Ratio_ as compared to *V*_RatioPerm_ imply that the UCM effects largely originated from multi-joint co-variation and not individual joint variability (Verrel 2011; Yen and Chang 2010). As for all UCM measures *V*_RatioPerm_ was log transformed before statistical analysis [*V*_RatioPerm_ = log(*V*_RatioPerm_)]. A detailed decription of the analysis can be found elsewhere (Greve et al. 2015; Verrel 2011; Yen and Chang 2010).

### Control experiment

To ascertain that age differences in motor flexibility were not due to adaptations in reaching kinematics and to establish how adaptations in reaching kinematics relate to motor flexibility, we performed a control experiment in 13 healthy young adults. The participants from the control experiment performed the same reaching task as in the main experiment, but were instructed to perform the reaching task at their preferred speed. The kinematic differences between the young adults from the main experiment and the young adults from the control experiment produced similar kinematic differences as between the young and old adults from the main experiment. By comparing motor flexibility between both young experiment groups we were able to establish how adaptations in end-effector kinematics affect motor flexibility. This was important to establish whether possible age differences in motor flexibility were the result from age-related differences in end-effector kinematics or age-differences in joint coordination patterns.

### Data analysis

We analyzed the data with MATLAB scripts (Version R2012, Natick, USA). The coordinate data of each marker were filtered using a bi-directional fourth-order Butterworth filter with a cutoff frequency of 8 Hz. Start and end of the movement was defined as the velocity of the pointer tip in forward direction above 2 mm s^−1^ and below 2 mm s^−1^. Based on the initiation and end of the movement the total movement time of each reaching trial was computed. Our UCM analysis was performed during the movement at 4 phases of the time normalized data and at the end-point of the reaching movement similar to a previous study (Greve et al. 2015). However, movement phase did not affect the results and we only present and discuss the results from the analysis at the end-point of the reaching movement. To compute the acceleration times, deceleration times and peak velocities of each reaching trial we used the tangential velocity of the pointer tip, computed based on the 3D position data. The sum of the square roots of the 3D position data gave the tangential end-effector position.

### Statistics

The statistical analysis was performed with SPSS 20.0. To investigate age differences in motor flexibility during rapid reaching we performed two repeated measures ANOVA. The first analysis was performed on *V*_Ratio_ with dexterity demand (ID 4 and ID 6) and physical demand (0 and 13%) as within-subject factors and age (young and old) as between-subject factor. The second analysis was performed on variability per DOF with UCM components (GEV and NGEV), dexterity demand, and physical demand as within-subject factor and age as between-subject factor. To establish age differences in end-effector kinematics five repeated measures ANOVA on total movement time, duration of the acceleration and deceleration phase, peak velocity and the effective target width with dexterity demand and physical demand as within-subject factor and age as between-subject factor were performed.

To determine whether UCM effects originated from individual joint variability or multi-joint co-variation and whether this differed between age groups, we performed a repeated measures ANOVA on variability per DOF with ratio component (*V*_Ratio_ and *V*_RatioPerm_), dexterity demand and physical demand as within-subject factor and age as between-subject factor.

We examined whether group differences in movement speed affected our findings on UCM measures with a repeated measures ANOVA on *V*_Ratio_ in the young adults from the main experiment and control group with dexterity and physical demand as within-subject factor and experiment group (control and main group) as between-subject factor. To establish the association between end-effector kinematics and motor flexibility in young and old adults we performed a repeated measures ANOVA on *V*_Ratio_ in the young and old adults from the main experiment group with dexterity and physical demand as within-subject factor, age as between-subject factor and total movement time, the duration of the deceleration phase and peak velocity values averaged across dexterity and physical constraints as co-variates (ANCOVA). Finally we investigated whether the duration of the deceleration phase was associated with the young and old adults’ GEV with correlation analysis. Therefore, we performed correlation analysis between GEV and the duration of the deceleration phase averaged across dexterity and physical constraints for both young experiment groups and the old adults separately.

If the assumption of sphericity was violated, the Greenhouse–Geisser correction was applied. To interpret the significant effects of the ANOVAs, the eta-squared (*η* ^2^) for effect size was used (Bakeman 2005; Olejnik and Algina 2003). The effect sizes were interpreted according to Cohen’s (Cohen 1988) recommendation of 0.02 for a small effect, 0.13 for a medium effect and 0.26 for a large effect (Bakeman 2005). Only statistically significant results (*p* < .05) and an *η* ^2^ value above 0.02 were further analyzed with post hoc comparisons.

Based on a previous study from Verrel et al. (2012; *η* ^2^ = 0.43) during which healthy young and old adults reached at their comfortable speed, we expected a moderate *η* ^2^ = 0.6 for a possible age effect on motor flexibility when reaching at maximum speed and under challenging task demands. We used G*Power and calculated that the required total sample size was 24 participants for an alpha level of 0.05 and a power of 0.8 (Faul et al. 2007).

## Results

### Participants’ characteristics

In total 29 (15 young and 14 old) participants performed 25 reaching trials for each experimental condition of which on average 21.3 ± 1.2 trials for each participant and condition were included in the analysis. Trials during which one or more markers were invisible were excluded from the analysis. Due to the larger number of females in both the healthy young and old adults group we performed two analyses to establish whether the gender affected the results. First we performed the repeated measures ANOVA on variability per degree of freedom (GEV and NGEV) in female participants only. In the second analysis we repeated this analysis with all participants included but with age and gender as between-subject factors. The results of the repeated measures ANOVA in only females revealed similar results as when all participants were. Furthermore, the repeated measures ANOVA with gender as between subjects factor did not reveal any significant main effects for gender or interaction effects between gender and variability (gender: *p* = .227, *F*_1, 25_ = 1.5; variability × gender: *p* = .736, F_1,25_ = 0.116). Therefore, we are confident that our results can be generalized to the general population.

Table 1 shows the anthropometrics, strength measurements, VAS-scores and clinical measurements for the young and old participants. Old compared to young participants had lower grip strength (*t*_21.2_ = 4.1; *p* < .01), performed slower on the 9 Hole Peg test (*t*_20.5_ = 5.3; *p* < .01), and had lower MVC (*F*_1, 27_ = 10.6; *p* = .003). The interaction effect between age and measurement suggests that the task was more fatiguing for the young as compared to the old adults (*F*_1, 27_ = 6.2; *p* = .019; *η* ^2^ = 0.16; Table 1). In line with the MVC measurements analysis of the VAS scores showed that the young adults perceived the experiment as more fatiguing (*t*_27_ = 3.8; *p* < .01; Table 1).

**Table 1:** Anthropometric, strength and clinical data

	Young		Old	
	Mean	±SD	Mean	±SD
Age (years)	22.5	2.3	75.4	5.2
BMI (kg/m^2^)	21.6	2.1	26.2	3.8
Body weight (kg)	67.0	9.1	74.6	9.5
Height (cm)	176.1	10.3	169	6.3
Strength (N)^a+^				
Pre	2.64	0.75	1.80	0.54
Post	2.43	0.60	1.81	0.54
Grip strength (kg)^a++^	0.44	0.09	0.29	0.06
9 Hole Peg test (s)^++^	16.9	1.9	22.4	3.4
VAS score^+^	36.3	16.2	17.4	9.3

^a^Strength profile normalized by body weight (kg); + indicates significant age difference (+ = *p* < .05; ++ = *p* < .01)

### Joint position data

**Fig. 2:**
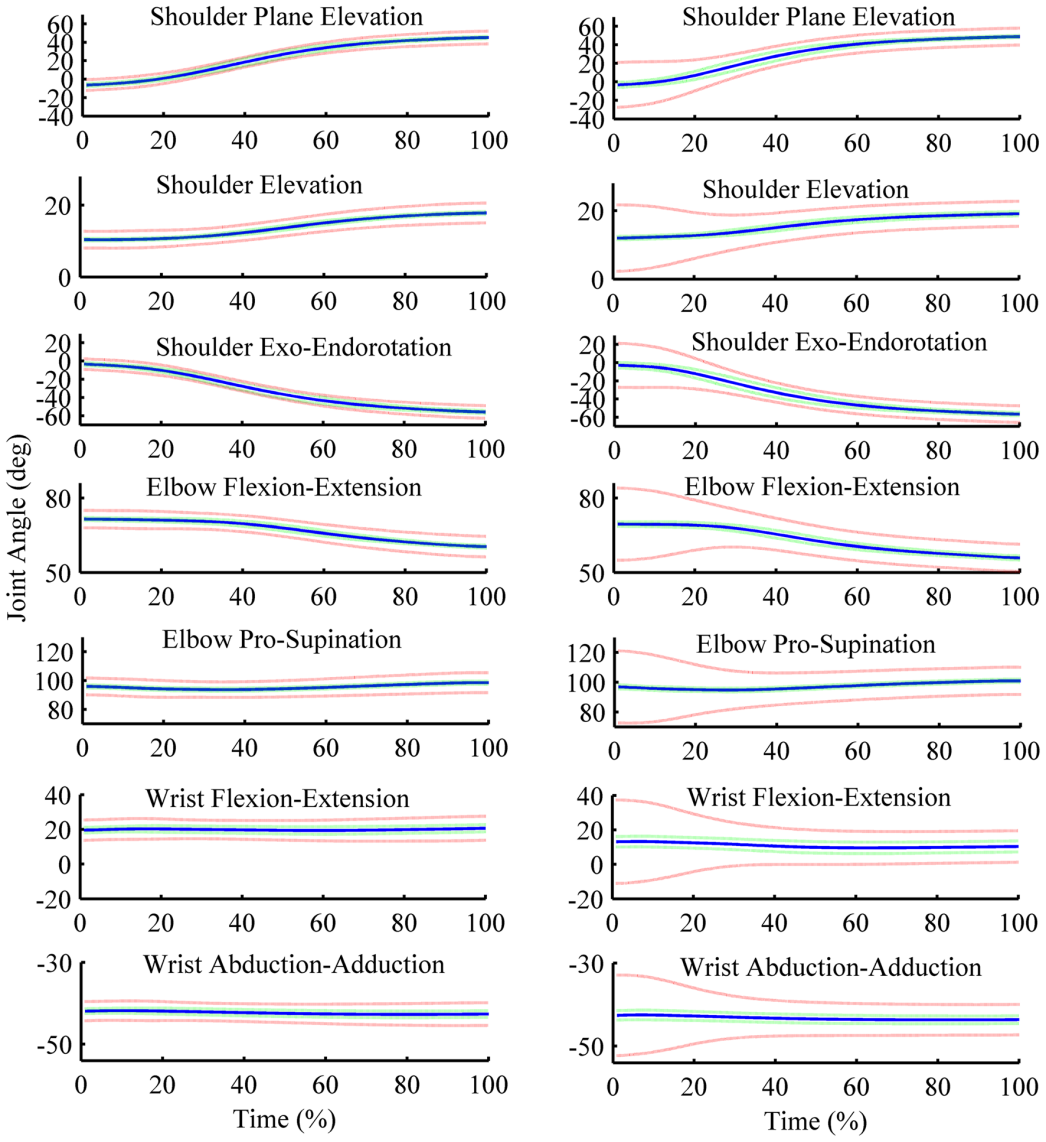
Time normalized joint position data of the ID 4 and 0-kg condition for young and old participants. The *blue line* gives the mean, the *dashed green line* gives the mean of the within participant standard deviation and the *red dashed line* gives the standard error of the mean of the time normalized joint position data in degrees of the shoulder, elbow and wrist joint. The *left panel* gives the time normalized joint position data of the young and the *right panel* of the old participants. (Color figure online)

Figure 2 shows the joint position data of the low dexterity and low physical demand conditions for young (left panel) and old (right panel) participants. Joint positions were similar between age groups, but the variation was greater in the old compared with the young group.

### Flexibility in motor behavior

**Fig. 3:**
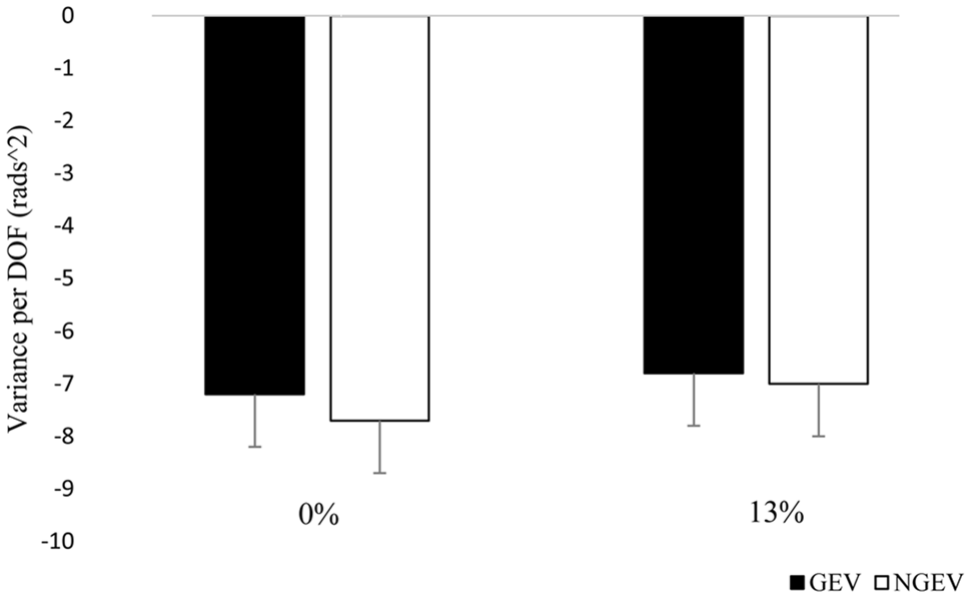
GEV and NGEV (log transformed) averaged across age and dexterity constraints for both physical demand conditions. *Vertical bars* denote standard error of the mean

Analysis on *V*_Ratio_ at the end-point of the reach did not reveal significant main or interaction effects. In addition to examining the effects of task constraints on the stabilizing effect of motor flexibility, we sought to determine whether variations in physical or dexterity constraints affected the variability components (GEV and NGEV) differently in the two age groups (Table 2). The repeated measures ANOVA revealed significant main effects for variability, physical demand and dexterity demand, but no interaction effects or significant age group effects. These findings are in line with our previous reaching experiment (Greve et al. 2015) and together with the results on *V*_Ratio_ demonstrate that both young and old adults similarly increased GEV and stabilized the *V*_Ratio_ as the amount of NGEV increased with increasing physical constraints of the reaching task (Fig. 3).

**Table 2:** Relevant main effects of repeated measures ANOVA on log transformed variability per DOF at the end-point of reaching for both male and female participants

Between/within-subject factor	Mean	SEM	*F*	*Df*	*p* value	*η* ^2^
Variability (end-point)						
GEV	−6.9	0.084	24.9	1, 27	<0.001	0.089
NGEV	−7.4	0.107				
Physical (end point)						
0%	−7.5	0.093	22.9	1, 27	<0.001	0.177
13%	−6.9	0.117				
Dexterity (end-point)						
ID 4	−7.1	0.090	6.6	1, 27	0.016	0.010
ID 6	−7.3	0.092				

### Individual joint variability and multi-joint co-variation

The permutation analysis aimed to determine whether UCM effects originated from multi-joint covariation or individual joint variability. Therefore, only the results pertaining to this question will be presented and discussed. The repeated measures ANOVA revealed a significant main effect for ratio component (*V*_RatioT_ = 0.41 ± 0.08, *V*_RatioPermT_ = 0.18 ± 0.05; *F*_1,27_ = 21.5, *p* = < 0.001, *η* ^2^ = 0.036) which implies that the UCM effects mainly originated from multi-joint covariation and not individual joint variability.

### Joint variability (coefficient of variation per joint angle)

The ANOVA to determine if old compared to young adults distributed the variability in the effector system differently across the involved joints during the reaching tasks, revealed significant main effects for physical demand and joint angle but no significant main or interaction effects for age group (Table 3). The main effect of physical demand showed that the total amount of variability in the effector system increases with the physical demand of the reaching task.

**Table 3:** Coefficient of variation at end-point of reaching movement

Within-subject factor	Mean	SEM	*F*	*df*	*p* value	*η* ^2^
Physical						
0%	0.038	0.002	56.1	1, 27	<0.001	0.135
13%	0.055	0.003				
Joint angle (rads)						
Shoulder plane elevation	0.053	0.003	54.5	3.6, 97	<0.001	0.349
Shoulder elevation	0.036	0.002				
Shoulder rotation	0.067	0.003				
Elbow flexion/extension	0.038	0.002				
Elbow pro-/supination	0.060	0.004				
Wrist flexion/extension	0.046	0.003				
Wrist ab-/adduction	0.025	0.002				

### End-effector kinematics

Table 4 shows the significant main and interaction effects from the repeated measures ANOVA on movement time, peak velocity and duration of the deceleration phase. The analysis revealed significant main effects for age, dexterity and physical demand on all kinematic measures and a significant interaction effect between age and physical demand for peak velocity values. The significant main effects for age showed that in line with previous reaching studies (Sleimen-Malkoun et al. 2013; Van Halewyck et al. 2015) old as compared to young adults moved slower, generated lower peak velocity values and prolonged the deceleration phase. The significant main effects for physical and dexterity demand demonstrate that movement time and the duration of the deceleration phase prolongs and peak velocity values decline for both old and young adults as the dexterity and physical constraints increase.

**Table 4:** Significant main and interaction effects of repeated measures ANOVA on end-effector kinematics for main and control experiment

	Between/within-subject factor	Mean	SEM	*F*	*df*	*p* value	*η* ^2^
Main experiment							
Movement time (s)	Age						
	Young	0.762	0.032	22.3	1, 27	<0.001	0.452
	Old	0.982	0.034				
	Dexterity						
	ID 4	0.541	0.034	68.5	1, 27	<0.001	0.293
	ID 6	0.576	0.036				
	Physical						
	0%	0.515	0.029	119.3	1, 27	< 0.001	0.436
	13%	0.547	0.035				
Peak velocity (m/s)	Age						
	Young	0.772	0.026	7.1	1, 27	0.013	0.207
	Old	0.672	0.027				
	Dexterity						
	ID 4	0.763	0.020	33.1	1, 27	<0.001	0.139
	ID 6	0.681	0.020				
	Physical						
	0%	0.805	0.019	191.0	1, 27	< 0.001	0.562
	13%	0.640	0.020				
	Physical × age			11.8	1, 27	0.002	0.035
Duration deceleration (s)	Age						
	Young	0.470	0.027	91.4	1, 27	< 0.001	0.541
	Old	0.686	0.027				
	Dexterity						
	ID 4	0.502	0.021	77.9	1, 27	<0.001	0.469
	ID 6	0.655	0.021				
	Physical						
	0%	0.535	0.019	32.1	1, 27	<0.001	0.150
	13%	0.621	0.022				
Control experiment							
Movement time (s)	Experiment group						
	Exp	0.751	0.038	96.4	1, 26	<0.001	0.788
	Control	1.296	0.041				
Peak velocity (m/s)	Experiment group						
	Exp	0.774	0.027	59.6	1, 26	<0.001	0.696
	Control	0.474	0.028				
Duration deceleration (s)	Experiment group						
	Exp	0.603	0.012	19.1	1, 26	<0.001	0.423
	Control	0.683	0.013				

The significant interaction effect between physical demand and age on peak velocity was further investigated with post hoc analysis on the 0 and 13% physical demand condition averaged across dexterity constraints. Post hoc analysis revealed that after Bonferroni correction old compared to young adults had significantly lower peak velocity values during the 0% physical demand condition (*t*_27_ = 3.7; *p* = .001), but not during the 13% physical demand condition (*t*_27_ = 1.5; *p* = .158).

### End-effector accuracy

**Fig. 4:**
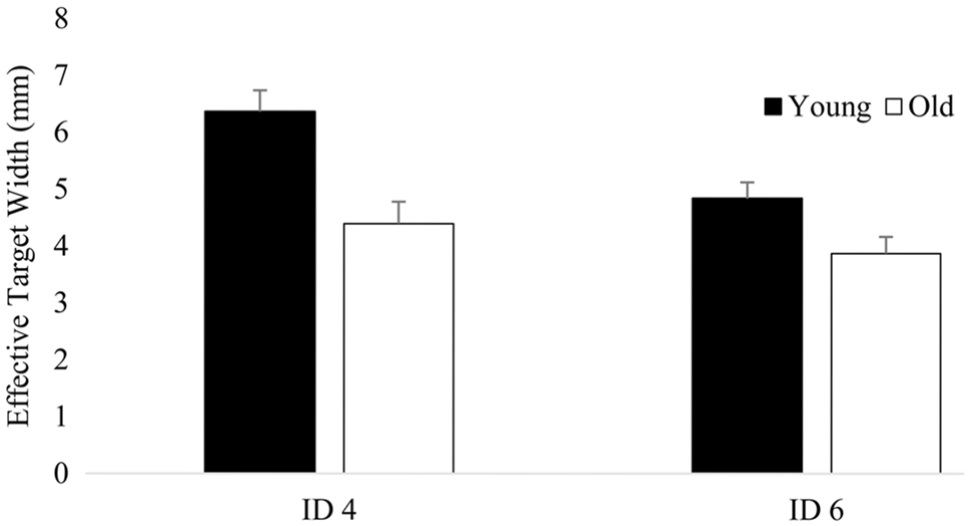
Effective target width for young and old participants and both dexterity demand conditions averaged across physical constraints. *Vertical bars* denote standard error of the mean

**Fig. 5:**
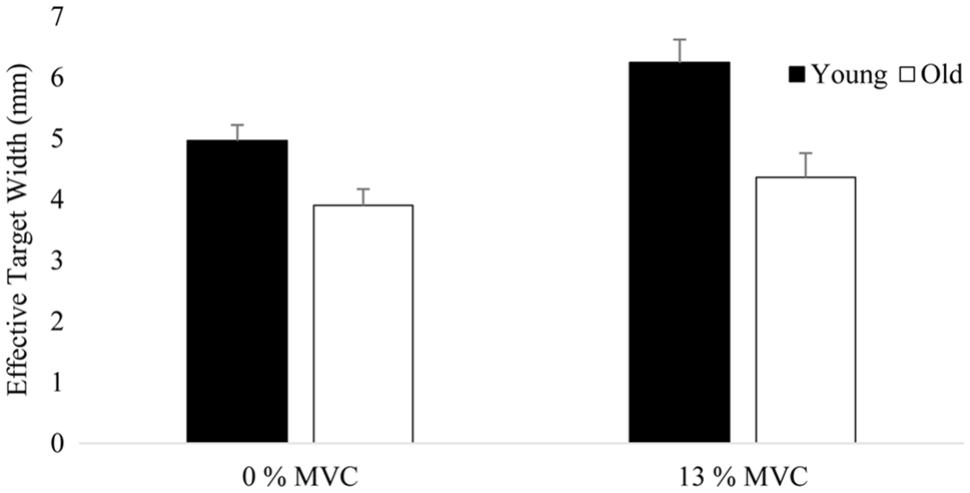
Effective target width for young and old participants and both physical demand conditions averaged across dexterity demand. *Vertical bars* denote standard error of the mean

Analysis of the effective target width of the pointer tip position revealed significant main effects for dexterity demand, physical demand and age and significant interaction effects between dexterity demand and age and dexterity demand and physical demand (Table 5; Figs. 4, 5). The main effect of age shows that the mean effective target width was smaller for the old as compared to young adults. Independent of the physical demand of the reaching task both young and old adults reached the large target (Young 6.4 ± 0.4 mm, Old 4.4 ± 0.4 mm ; ID 4 = 13.9 mm), but sometimes missed the small target (Young 4.8 ± 0.3 mm, Old 3.9 ± 0.3 mm; ID 6 = 3.9 mm) when reaching as fast as possible (Fig. 4). As illustrated in Fig. 4 the significant interaction effect between dexterity demand and age implies that the effective target width declined more in the young as compared to old adults when reaching to the small as compared to the large targets (Fig. 4). Figure 5 shows that the effective target width increased to a similar extent in the young and old adults during the high as compared to low physical demand conditions (Fig. 5).

**Table 5:** Significant main and interaction effects of repeated measures ANOVA on the effective target width

Within/between-subjects factor	Mean	SEM	*F*	*df*	*p* value	*η* ^2^
Age						
Young	5.602	0.291	12.3	1, 27	0.002	0.312
Old	4.134	0.301				
Physical						
0%	4.433	0.186	17.6	1, 27	<0.001	0.131
13%	5.303	0.276				
Dexterity						
ID 4	5.381	0.267	21.0	1, 27	<0.001	0.182
ID 6	4.335	0.205				
Dexterity × age			5.0	1, 27	0.034	0.043
Dexterity × physical			5.0	1, 27	0.034	0.028

### Associations between end-effector kinematics and motor flexibility

The repeated measures ANOVA on the *V*_Ratio_ of both young groups from the control and main experiment did not reveal any significant main or interaction effects between experiment groups and *V*_Ratio_. Similarly, the ANCOVA in the young and old participants revealed that none of the investigated covariates were significantly associated with the young or old adults’ *V*_Ratio_. Also the correlation analysis showed that there were no significant correlations between the duration of the deceleration phase and GEV in the young or old adults from the main experiment (Young: *r* = − .296, *p* = .303; Old: *r* = .404, *p* = .135) and the young adults from the control experiment group (*r* = − .015, *p* = .960). In summary, we could not identify an association between end-effector kinematics and the young and old adults’ motor flexibility.

## Discussion

The current study had two goals: (1) to determine the effects of age on the use of the available motor flexibility while performing goal-directed reaching under physical and dexterity constraints and (2) to examine the association between end-effector kinematics (i.e., reaching speed) and motor flexibility in each age group.

Our findings demonstrated that age does not affect motor flexibility although healthy young and old adults performed the reaching task under high physical and dexterity demands. Both age groups were similarly able to compensate for larger NGEV with increasing physical demands by increasing the available range of those motor solutions stabilizing the end-effector position (GEV). This proportional increase in GEV allowed participants to maintain performance stability (*V*_Ratio_) despite larger de-stabilizing variability when performing fast but accurate reaching tasks under high physical demands. Dexterity demand did not affect motor flexibility. We further showed that end-effector kinematics did not correlate with motor flexibility.

### Healthy ageing and a seemingly paradoxical preservation of motor flexibility

Considering the age-related decline in neuromuscular function, our finding that healthy young and old adults employ similar motor flexibility might be somewhat unexpected. Indeed, old compared with young adults have deficits in muscle strength (Faulkner et al. 2007; Thompson 2009), muscle power (Bassey et al. 1992; Faulkner et al. 2007; Thompson 2009) and mobility (Beijersbergen et al. 2013), are less able to integrate proprioceptive feedback (Goble et al. 2009) and to coordinate agonist–antagonist muscle pairs (Hortobágyi and Devita 2006), critical in reaching movements. Furthermore, old adults show decrements in central nervous system functioning such as a reduction in motor cortical inhibition (Hortobágyi et al. 2006; Papegaaij et al. 2014; Peinemann et al. 2001), white matter lesions (Ge et al. 2002; Pantoni 2002; Schulz et al. 2014) and decrements in the number and size of afferent fibers (Romanovsky et al. 2015). Such neuronal and neuromuscular deficits have been associated with impaired and slow execution of ADLs (Rosano et al. 2012; Sleimen-Malkoun et al. 2013; Van Halewyck et al. 2015), poor balance control (Baloh et al. 2003; Huxhold et al. 2006; Papegaaij et al. 2014) and mobility disability in walking (Beijersbergen et al. 2013; Rosano et al. 2012; Sorond et al. 2015). Despite such age-related deficits, there is inconclusive evidence as to how and if at all advancing age affects motor flexibility during multi joint tasks (Greve et al. 2013, Hsu et al. 2013, 2014; Krishnan et al. 2013; Krüger et al. 2013; Olafsdottir et al. 2007; Skm et al. 2012; Verrel et al. 2012; Xu et al. 2013). Comparing old vs. young adults, Verrel et al. (2012) reported poorer motor flexibility in a horizontally directed reaching task, whereas Krüger et al. (2013) reported greater motor flexibility in a forward reaching task, and Xu et al. (2013) found similar motor flexibility in a reaching assembly task. Our findings extend these data by demonstrating an absence of age effect on motor flexibility during rapid, goal-directed reaching even when performed under challenging task constraints (Table 2; Fig. 3). In sum, these data suggest a seemingly paradoxical preservation of motor flexibility in healthy old adults and that healthy ageing affects end-effector kinematics independent of motor flexibility during rapid reaching.

Our finding that motor flexibility is preserved in old adults’ reaching behavior can be supported by studies investigating old adults’ adaptation capacity during reaching (Bock 2005; Buch et al. 2003; Cressman et al. 2010; Heuer and Hegele 2008). These studies examined whether or not old adults can restore reaching accuracy after a visual perturbation. For example, there was a lack of age effect on reaching performance in a virtual environment when the cursor misrepresented the position of the hand through a rotation in the visual field (Buch et al. 2003). Analysis of end-effector performance showed that old as compared to young adults were less able to restore performance accuracy when the distortion in the visual field was introduced abruptly (Bock 2005; Heuer and Hegele 2008) but equally able when the distortion was introduced gradually (Buch et al. 2003; Cressman et al. 2010). The gradual perturbation consisted of a rotation in the visual field of 0.75 degrees clockwise after the initiation of the reaching movement until a total of 30 degrees was reached within 41 repetitions (Cressman et al. 2010). Placing these data in the context of our results, we argue that the old adults’ ability to gradually adapt to visuomotor perturbations during reaching is due to their preserved ability to flexibly choose an adequate motor solution from a large range of available joint configurations to perform the same reaching movement. In sum, the results from our experiment combined with previous data (Buch et al. 2003; Cressman et al. 2010) suggest that healthy aging does not affect motor flexibility during goal-directed reaching tasks.

### Motor flexibility and end-effector kinematics

Our findings showed that healthy young and old adults can modulate reaching kinematics independent from motor flexibility. While in the main experiment participants reached at maximal speed, in the control experiment we matched the two age groups’ reaching speed and compared motor flexibility. Young adults in the control vs. the main experiment reached more slowly, achieved lower peak velocities and longer deceleration times, mimicking the effects of age on end-effector kinematics as reported previously and observed in the main experiment (Sleimen-Malkoun et al. 2013; Van Halewyck et al. 2015). We found no difference in motor flexibility between the young groups reaching rapidly or at a self-selected pace, which implies that movement speed did not affect our motor flexibility data. Predictably, age-related adaptations in end-effector kinematics did not correlate with adaptations in motor flexibility. Thus, movement speed did not confound the motor flexibility data. Furthermore, there was no association between the kinematic behavior and motor flexibility in young and old adults implying that the neuromuscular system can adapt end-effector kinematics independent of motor flexibility and vice versa. We propose that the ability to independently modulate reaching kinematics and motor flexibility is advantageous because young and old adults alike can perform goal-directed reaching tasks rapidly without compromising motor flexibility.

### Limitations

We suspect that our and previous experimental setups might limit young and old adults’ capacity to explore the available motor flexibility, leaving possible age differences undetected during reaching. The UCM method decomposes trial-to-trial variability in the effector system into variability stabilizing and de-stabilizing the performance variable around its mean (Latash et al. 2002, 2007; Scholz and Schöner 1999). This across trial analysis requires participants to repeatedly execute the movement from the same start to the same target position. This experimental setup and design (i.e., the repetitive movement from one position to another) might confine the exploration of alternative joint angle configurations. The restricting nature of the setup might have minimized any age effects on exploratory behavior even when participants performed the task as fast as possible under challenging dexterity and physical demands. We, once again, note that a lack of age effect on flexibility was present vis-à-vis the low acceleration and prolonged deceleration of the reaching movement in old adults, a finding reported previously (Sleimen-Malkoun et al. 2013; Jacques et al. 2013). Lastly, most of our participants were female and even though the statistical analyses suggested no gender effects, it is still possible that gender inequality might have affected the results.

### Conclusion

The data challenge the prevailing view that old age affects movement capabilities in general and provide evidence that healthy old adults preserve motor flexibility during a goal-directed upper extremity reaching task. We propose that a preservation of motor flexibility would allow both young and old adults to accurately perform reaching tasks under various physical and dexterity constraints. Future studies should examine the underlying mechanisms of motor flexibility. Studies applying UCM analysis should investigate how experimental set-ups affect movement exploration, leaving possible age differences undetected.

## Abbreviations

ADL: Activities of daily living
ANOVA: Analysis of variance
ANCOVA: Analysis of co-variance
DOF: Degrees of freedom
EMG: Electromyography
GEV: Goal-equivalent variability
ID: Index of difficulty
J: Jacobian
MVC: Maximal voluntary contraction
NGEV: Non-goal-equivalent variability
STD: Standard deviation
UCM: Uncontrolled manifold
VAS: Visual analogue scale
